# Structure-resistive property relationships in thin ferroelectric BaTiO$_{3}$ films

**DOI:** 10.1038/s41598-020-72738-5

**Published:** 2020-09-28

**Authors:** N. V. Andreeva, A. Petraru, O. Yu. Vilkov, A. E. Petukhov

**Affiliations:** 1 St. Petersburg Electrotechnical University ‘LETI’, Saint Petersburg, Russia 197376; 2 Nanoelektronik, Technische Fakultät, Christian-Albrechts-Universität zu Kiel, 24143 Kiel, Germany; 3 Department of Solid State Electronics, St. Petersburg State University, Saint Petersburg, Russia 198504; 4 Research Park, St. Petersburg State University, Saint Petersburg, Russia 198504

**Keywords:** Electronic properties and materials, Ferroelectrics and multiferroics, Surfaces, interfaces and thin films

## Abstract

A combined study of local structural, electric and ferroelectric properties of SrTiO$_{3}$/La$_{0.7}$Sr$_{0.3}$MnO$_{3}$/BaTiO$_{3}$ heterostructures was performed by Piezoresponse Force Microscopy, tunneling Atomic Force Microscopy and Scanning Tunneling Microscopy in the temperature range 30–295 K. The direct correlation of film structure (epitaxial, nanocrystalline or polycrystalline) with local electric and ferroelectric properties was observed. For polycrystalline ferroelectric films the predominant polarization state is defined by the peculiarity of screening the built-in field by positively charged point defects. Based on Scanning Tunneling Spectroscopy results, it was found that a sequent voltage application provokes the modification of local resistive properties related to the redistribution of point defects in thin ferroelectric films. A qualitative analysis of acquired Piezoresponse Force Microscopy, tunneling Atomic Force Microscopy and Scanning Tunneling Microscopy images together with Scanning Tunneling Spectroscopy measurements enabled us to conclude that in the presence of structural defects the competing processes of electron injection, trap filling and the drift of positively charged point defects drives the change of resistive properties of thin films under applied electric field. In this paper, we propose a new approach based on Scanning Tunneling Microscopy/Spectroscopy under ultrahigh vacuum conditions to clarify the influence of point defects on local resistive properties of nanometer-thick ferroelectric films.

## Introduction

Since the first experimental observations of resistive switching effect in ultrathin ferroelectric (FE) layers^1,2^ were allowed by advances in FE films technology, a research interest in the respective structures has been rising rapidly owing to their potential electronic applications. For instance, FE tunnel junctions (FTJs)—capacitors with a nm-thick FE barrier layer are considered to be efficient as non-volatile memory cells, competing there with complementary metal-oxide-semiconductor (CMOS) technologies. Some key advantages of FTJs are non-destructive read-out process, ultra-low power consumption, high storage density, *etc*. In FTJs structures, complex oxides are usually used as the bottom electrodes and pure metals as the top ones. Due to the difference in the effective screening lengths of the electrodes, an asymmetry in the electrostatic potential in FTJs alters the mean barrier height for two opposite polarization orientations in thin FE film^3^ and thus, this mechanism seems to govern the resistance switching in FTJs. Theoretically predicted variation of tunneling electro-resistance for commonly used materials could not exceed one or two orders of magnitude. At the same time, experiments showed that in some cases it was possible to tune electro-resistance by 4–5 orders of magnitude^4^ by changing the amplitude, the duration or the number of voltage pulses applied to the top electrode (TE) of the FTJ structure^5–7^.

A closer look at the details of the experiments reveals the following key points. First, FE film thickness *d* in the FTJ structures reported in some works, e.g., 4.6 nm^7^, assumes that besides an elastic tunneling, a dominant transport mechanism in FTJs, there should be another contribution to the electron current across the structure^8^. Next, the value of the applied voltage, at which different resistance states appear, significantly exceeds the coercive field of FE thin film^6^, suggesting a rather complex way of the resistance changing in the FTJ structures. Finally, a strong impact of charged oxygen vacancies migration on the resistive switching in thin FE films was experimentally observed recently^9–11^. In that regard, in FE films with a thickness greater than the mean free path of the injected electrons, it is reasonably to suppose that the point defects start playing an important role. As a result, polarization state effects combined with an inelastic trap-assisted tunneling would enable a hybrid resistive switching mechanism in FE films with point defects involved. These latter contribute both to the electron transport and to the screening of the polarization charges, and the contribution becomes significant when the thickness of the film exceeds a certain value, below which an elastic tunneling through the barrier prevails in electron transport. Strictly, these rather thick structures could not be considered as FTJs anymore.

An important issue concerns type and density of point defects, ensuring their influence on the resistive properties of FE films. In perovskite oxides with no acceptor dopants, point defects are considered to be mainly related either to oxygen vacancies, which can be neutral $\hbox {V}_{\mathrm{o}}$, singly $\hbox {V}_{\mathrm{o}}^{+}$ and doubly positive $\hbox {V}_{\mathrm{o}}^{\mathrm{2+}}$, or to $\hbox {V}_{\mathrm{o}}$-related complexes, e.g., $\hbox {Ti}^{\mathrm{3+}}-\hbox {V}_{\mathrm{o}}$ centers^12,13^. There are studies that assigned the conductivity in oxygen-deficient FE films to the hopping of mobile oxygen vacancies^14,15^. However, the following question is still open: At what densities of oxygen vacancies do they contribute sufficiently to the conductivity and to the resistive switching? As we consider, the required densities are not achievable in epitaxial FE films with a given cation stoichiometry and with a thickness, at which the polarization state correlates with the resistive switching. Our estimation based on experiments with thin metal-oxide films^16^ gives an approximate value of $10^{19} {\mathrm{cm}}^{-3}$ for the density of oxygen vacancies. Thus, replacing the epitaxial FE film by the polycrystalline one seems to be necessary to spot the effects caused by the vacancies. Then, varying the deposition parameters of the film, such as temperature, chemical potential (oxygen partial pressure) and relaxation time of point defect equilibria (thermal history), one may tune the density of point defects to reveal their role in the resistive switching phenomena.

Here, we study the influence of FE film structure on the charge transport properties and resistive switching effects in thin FE films in the temperature range of 30–295 K. We propose an approach, which includes scanning tunneling microscopy/spectroscopy (STM/STS) measurements under ultrahigh vacuum (UHV) conditions. Similar to tunneling atomic force microscopy (tAFM), widely used for local electrical measurements, STM/STS are the surface-sensitive methods, which allow us to study local electronic structure of an active FE layer with a higher spatial resolution on subnanometer-scale^17,18^. UHV conditions ensure the adsorbate-free sample surface, which is necessary to avoid the charge screening of the active layer and to increase the reliability of the experimental data. Successfully applying STM, we have studied the local resistive and FE properties of SrTiO$_3$/La$_{0.7}$Sr$_{0.3}$MnO$_3$/BaTiO$_3$ (STO/LSMO/BTO) structures with epitaxial and polycrystalline FE films. The results obtained with STM are found to sufficiently augment the tAFM measurements.

## Results and discussion

**Figure 1:**
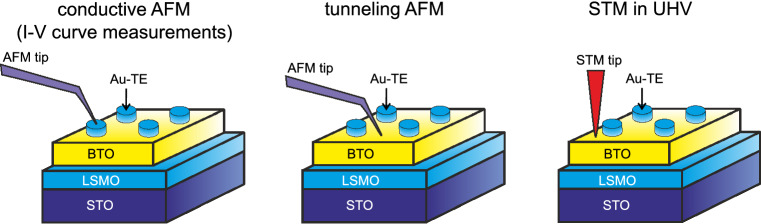
Structure of the samples under study and schematic illustration of the methods for their morphological and resistive properties investigation. Left-to-right: conductive AFM with a tip placed on top of gold electrodes, tunneling AFM and STM under UHV conditions on bare BTO films.

Figure 1 illustrates the proposed approaches to study morphological and resistive properties of FE films. Deposited on sample surface, TEs are used for *I*–*V* curve measurements with conductive AFM technique. Pt-coated silicon AFM tip is placed on top of the gold platelets, and *I*–*V* characteristics are measured by applying the quasi-static voltage sweeps. Such an approach helps to eliminate a specific influence of a contact between the AFM tip and the sample on the obtained results. However, a micrometer-size metallic electrode does not provide the required locality of the measurements. Moreover, in case of thin FE films, a metallic TE significantly changes the screening conditions of the polarization charges, influencing the electric and FE properties. Local resistive characteristics are usually measured on the bare film surfaces with tAFM, implying ultra-low-current measurements from poor conductors. In these experiments, conductive tip serves as TE and a feedback control in AFM relies on Van-der-Waals force interaction. Unfortunately, temperature induced variation of a cantilever k-constant reduces the reliability of T-dependent measurements carried out with tAFM^19^. The impact of electrostatic forces between the AFM cantilever and the surface of a dielectric film, and a reduced contact area due to the absence of an adsorbate layer on the sample surface under UHV conditions are of special importance among other points to be kept in mind for the tAFM measurements. To overcome these issues, STM technique is proposed here to study morphological and local resistive properties of FE films. Tunnel current, as a feedback control parameter in STM, provides stable, temperature independent probe-sample contact, in contrast to force interaction used in a feedback loop of AFM. Also, compared to AFM, STM ensures better spatial resolution of the measured characteristics. Set points for the tunnel current $\hbox {I}_{\mathrm{t}}$ and the voltage $\hbox {V}_{\mathrm{s}}$ between the STM tip and the bottom electrode should be chosen in accordance with the resistive properties of the film. The TEs have been used in this work only for *I*–*V* measurements with the AFM tips.

Let us now briefly describe the morphological peculiarities of the grown samples, and then we go through the detailed analysis of their local FE and resistive properties. As is mentioned above, varying the parameters of pulsed laser deposition (PLD), such as temperature, pulse energy, oxygen pressure, etc., we are able to control the structure of BTO films. Among our samples are high-quality epitaxial and polycrystalline films with variable grain sizes. The growth conditions are listed in Table S1 of Supplementary materials. The samples for the current studies have been chosen as follows. To a first approximation, formation of point defects is mainly attributed to the density of grain boundaries in the films deposited at similar conditions and exhibiting comparable electric characteristics. The grain boundary density can be evaluated through the volume-to-surface ratio ($\mathrm{V/S}$) of a single grain. Considering the shape of the grain as a spherical segment, we can draw the following equation: $\mathrm{V/S}=\mathrm{h/2}\cdot \left( 1-h/3R\right)$, where $\mathrm{h}$ is a film thickness, and $\mathrm{R}$ is an equivalent disk radius of the grain. If $\mathrm{h<R}$, $\mathrm{V/S}$ increases with increasing $\mathrm{R}$, whereas densities of grain boundaries and point defects diminish. Bearing this in mind, we studied polycrystalline samples different sufficiently from each other in grain sizes. Also, we compared these films with epitaxial one, taking the local electric and FE properties of the latter as reference.

**Figure 2:**
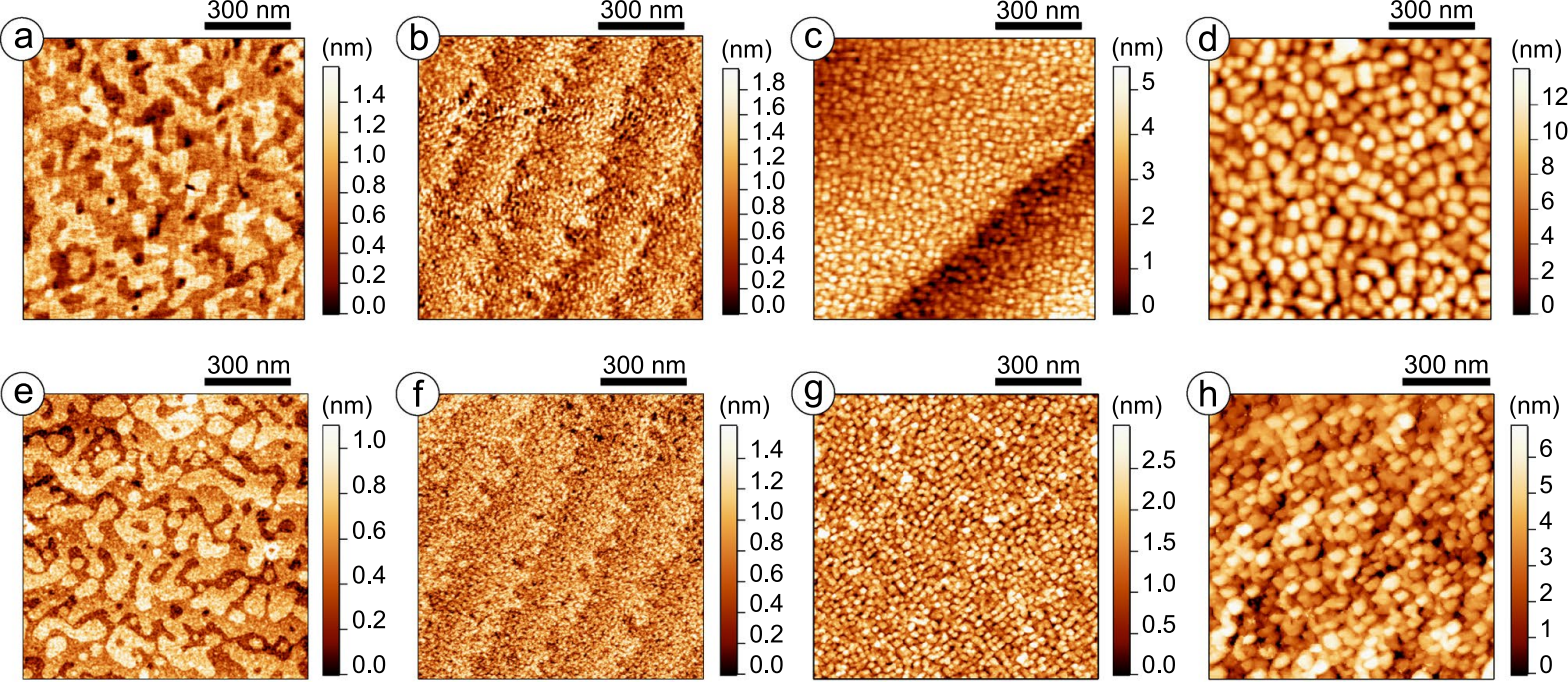
Scanning probe microscopy investigation of surface morphology. Contact mode AFM images of the BTO film surfaces: (**a**) epitaxial, (**b**) nanocrystalline, and (**c**), (**d**) polycrystalline with medium and large crystallites, correspondingly. STM images of the same BTO film surfaces: (**e**) epitaxial film from (**a**) ($\hbox {V}_{\mathrm{s}}=4.5$ V, $\hbox {I}_{\mathrm{t}}=1$ pA), (**f**) nanocrystalline film from (**b**) ($\hbox {V}_{\mathrm{s}}=4$ V, $\hbox {I}_{\mathrm{t}}=10$ pA), (**g**, **h**) polycrystalline films with different sizes of crystallites from (**c**) and (**d**), accordingly ($\hbox {V}_{\mathrm{s}}=1.5$ V, $\hbox {I}_{\mathrm{t}}=10$ pA and $\hbox {V}_{\mathrm{s}}=2$ V, $\hbox {I}_{\mathrm{t}}=10$ pA, accordingly).

**Figure 3:**
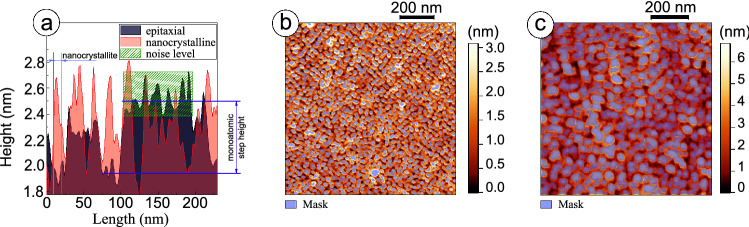
Statistical analysis of film surface morphology: (**a**) correlation of the profiles taken from the images of epitaxial and nanocrystalline films (Fig. 2a,b), (**b**) the results of grain selection procedure for polycrystalline film with medium grains (Fig. 2c), (**c**) the results of grain selection procedure for polycrystalline film with large grains (Fig. 2d).

Figure 2 shows morphology maps obtained with contact AFM at ambient conditions and STM under UHV for epitaxial (Fig. 2a,e) and polycrystalline films with variable grain sizes: nanocrystalline (Fig. 2b,f), polycrystalline with medium (Fig. 2c,g) and large (Fig. 2d,h) grains. The surface of epitaxial BTO film consists of two-dimensional atomically smooth, unit-cell-height islands attributed to the incomplete upper layer of the film under the step-flow growth conditions^20^. In the images of nanocrystalline film, clearly distinguished are the steps of monoatomic height formed on vicinal surface of single crystalline STO substrate. The detailed analysis reveals the lateral size of the grains for nanocrystalline film to be a few nanometers, that is comparable with a film thickness, and the height of the grains to exceed the unit cell size in the c-axis direction of barium titanate (Fig. 3a). Note, the unit cell sizes were determined in XRD experiments with epitaxial films, see Supplementary materials, Fig. S2. The root mean square (RMS) values of the grain height were estimated from the $1\times 1~\upmu {\mathrm{m}}^2$ images for epitaxial and nanocrystalline films and were found to be $1.57\pm 0.20$ nm and $2.16\pm 0.27$ nm, correspondingly. It should be mentioned that the deposition conditions for these two samples are comparable.

To determine $\mathrm{V/S}$ ratio for the two remaining polycrystalline samples, statistical analysis of the AFM images was applied including the following grain parameters: equivalent disk radius ($\hbox {r}_{\mathrm{eq}}$), total boundary length ($\hbox {L}_{\mathrm{tot}}$) and RMS value of the height. For polycrystalline film with medium grains (Figs. 2c,g, 3b), analysis of 1777 grains gives $\hbox {r}_{\mathrm{eq}}=6.48\pm 3.51$ nm, $\hbox {L}_{\mathrm{tot}}=90~\upmu {\mathrm{m}}$, and 0.73 nm for RMS value of the height. For polycrystalline film with large grains (Figs. 2d,h, 3c), corresponding values evaluated on 353 grains are $18.61\pm 10.69$ nm, $51~\upmu {\mathrm{m}}$, and 0.93 nm. Thus, three times reducing the lateral grain sizes means roughly doubling the total boundary length.

Complementary STM measurements (Fig. 2e–h) give evidence for the correlation between the morphology and electrical properties of BTO films. The surface corrugation is similar in AFM and STM, there are no protrusion or depression features related to the differences in local conductivity. The results of STM measurements in UHV justify the validity of this technique for local electrical property investigation of thin FE films. Note that the epitaxial and the nanocrystalline films require higher $\hbox {V}_{\mathrm{s}}$ than the polycrystalline ones for scanning at the same level of $\hbox {I}_{\mathrm{t}}$.

**Figure 4:**
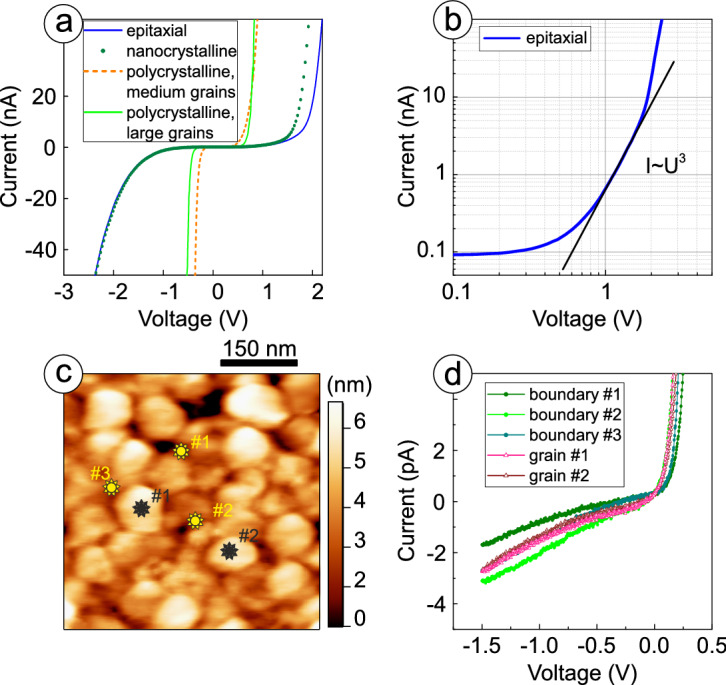
Resistive properties of STO/LSMO/BTO heterostructures: (**a**) *I*–*V* characteristics of epitaxial and polycrystalline films with different grain sizes measured with conductive AFM tip over the TE, (**b**) plotted in a double logarithmic scale *I*–*V* characteristic typical for epitaxial and nanocrystalline films measured with conductive AFM tip over the TE, (**c**) STM image of polycrystalline film with large grains ($\hbox {V}_{\mathrm{s}}=2$ V, $\hbox {I}_{\mathrm{t}}=10$ pA), (**d**) local *I*–*V* characteristics of same polycrystalline film measured with STS at different locations specified in (**c**).

To find a relation between the structural and the electric properties of FE films, we firstly analyze *I*–*V* characteristics measured with conductive AFM tip over the gold TEs, see Fig. 4a. *I*–*V* curves of epitaxial and nanocrystalline films are rather similar: they are nonlinear and symmetrical with regard to the polarity of the bias applied. The resistivity of FE films can be estimated from the linear part of *I*–*V* curves reflecting the case when the number of thermally generated carriers in the film exceeds the number of carriers injected from the electrode. For the structures with epitaxial and nanocrystalline BTO films and 36 $~{\upmu {\mathrm{m}}^{2}}$-sized TEs, the current is below 1 pA in a linear part of *I*–*V* curves, giving thus a resistivity of $10^{12}\Omega {\cdot}\mathrm{cm}$, at least. Plotted in a double logarithmic scale (Fig. 4b), *I*–*V* curves of these samples exhibit linear parts corresponding to the power dependences $I{\sim }U^n$ with $n=3$. This is typical for trap-assisted inelastic tunneling (TAT) influenced by space charge limited current (SCLC)^21^. Similarity of the electric properties of epitaxial and nanocrystalline films is related to the low concentration of point defects in these samples, which act as trap centers and provide the dominant transport mechanism (SCLC and TAT). For the samples with medium and large grains, *I*–*V* characteristics measured over TEs differ considerably from epitaxial and nanocrystalline films. They are nonlinear and asymmetric with regard to the polarity of the voltage applied, indicating the appearance of the rectifying barrier at the interface with the film. The resistivity of polycrystalline films estimated from the linear part of *I*–*V* curves is in the bottom of $10^{12}\Omega {\cdot} {\mathrm{cm}}$ range.

**Figure 5:**
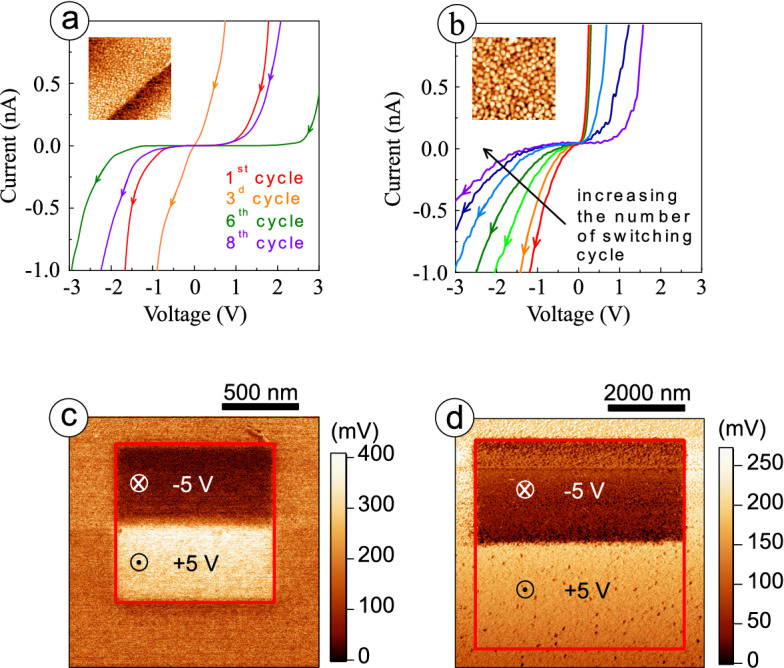
Cycling STS measurements of *I*–*V* characteristics for polycrystalline films at $\mathrm{T}=295$ K: (**a**) with medium grains, (**b**) with large grains. PFM phase images after the poling procedure: (**c**) for nanocrystalline film, (**d**) for polycrystalline film with large grains. Square domains with half squares of downward and upward polarizations written with $\pm 5$ V.

To check the uniformity of point defect distribution, we studied the resistive properties of both boundaries and bulk of a grain with local STS measurements. Since no statistically reliable differences are found in these experiments (Fig. 4c,d), $\mathrm{V/S}$ ratio is assumed to correlate well with the point defect density in polycrystalline films, and the defects are considered to be mainly related to the oxygen vacancies. With a bias voltage applied, the migration barrier lowers for the positively charged oxygen vacancies^22^. Moreover, the activation energy for this migration also reduces with increasing density of the defects^23–25^. This all together enables redistribution of the oxygen vacancies over the film thickness under applied electric field in polycrystalline samples. Such a process is accompanied by the change of the resistance state of FE film with TAT being a prevailing charge transport mechanism: probability of tunneling strongly depends on the variation in distance between the traps. This can be observed through the change of the slope in the linear part of *I*–*V* curve under cycling STS measurements, when quasi-static voltage sweeps are applied at same location on the surface, see Fig. 5a,b. In our experiments, acquisition and raster time at each point of *I*–*V* curve were 19.7 ms and 20 ms, respectively, with 100 points in a single voltage sweep in one direction.

The resistance state of polycrystalline films is found to vary in the range of one order of magnitude. The main difference between the samples with medium and large grains appears in the character of the transition from one resistive state to another. For FE film with medium grains, i.e. at smaller $\mathrm{V/S}$ ratio, the resistance state between two sequent sweeps changes stochastically (Fig. 5a), while the transition is gradual for the sample with larger $\mathrm{V/S}$ ratio (Fig. 5b). Establishing a new resistance state at cycling measurements is considered to be influenced mainly by a combination of two processes: redistribution of the point defects under applied electric field and their back-diffusion in the delay between sequent voltage sweeps. Decreasing density of the point defects, i.e. increasing $\mathrm{V/S}$ ratio, enlarges the activation energy for the defect migration, that, in turn, reduces diffusion coefficient. As a result, the role of the back-diffusion diminishes, and the gradual change of the resistance state for the sequent voltage sweeps is observed in case of film with large grains.

Local FE properties and their correlation with film morphology were investigated with piezoresponse force microscopy (PFM), see Figs. 5c,d and Fig. S5 of Supplementary materials. Epitaxial, nanocrystalline and polycrystalline samples with medium grains, comparable in size to the film thickness, exhibit a uniform piezoresponse distribution as well as symmetric and rectangular FE loops, see Supplementary materials for details (Fig. S5). The results of the poling procedure, that is a scan over rectangular regions with DC voltage between the bottom electrode and the grounded conductive tip, reflect the fact, that changing the polarity of the voltage applied gives rise to the corresponding polarization reorientation in the film: bright and dark areas in the phase images in Fig. 5c are related to the upward or downward direction of polarization, respectively. For films with large crystallites, FE domain structure is clearly distinguished inside individual grains in the as-grown state. Prevalence of the domains with an upward polarization (60–70% of the total number of domains) is observed. A significant contrast in the PFM phase distribution (Fig. 5d) appears only after poling with negative voltage and is attributed to the downward polarization orientation. The image of the regions poled with positive voltage does not differ considerably from that of the pristine state. Together with local piezoresponse loops shown in Supplementary materials (Fig. S5f), these results justify the preference of an upward polarization orientation in as-grown polycrystalline BTO films with large grains. The mean value of the imprint bias is about 0.4 V for polycrystalline film with large grains. The shape of the local hysteresis loop does not change much at different locations, indicating a uniform distribution of the local FE properties over the film surface.

**Figure 6:**
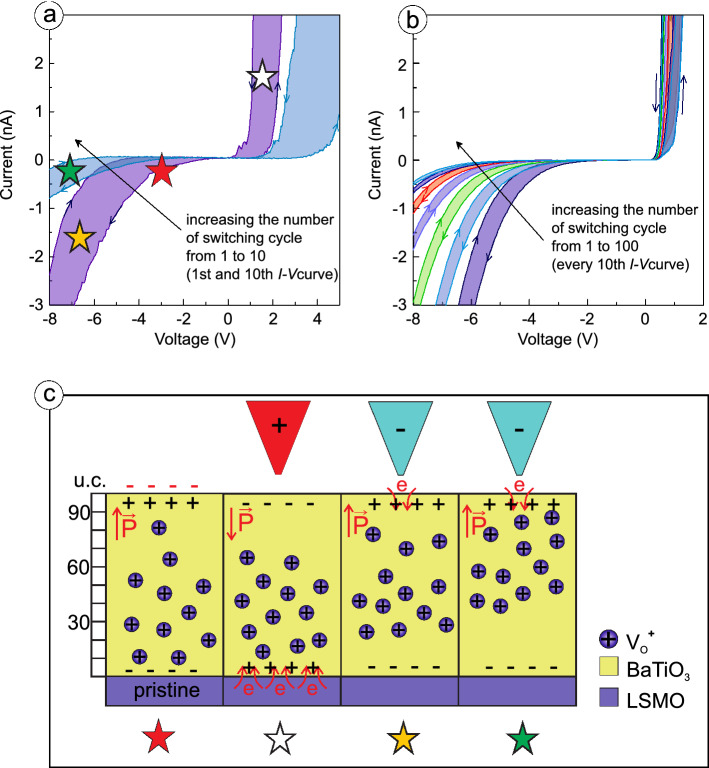
Cycling STS measurements of *I*–*V* characteristics for polycrystalline film with large grains: (**a**) at $\mathrm{T}=295$ K, and (**b**) at $\mathrm{T}=30$ K. (**c**) schematic illustration of the impact of point defects distribution on the resistive properties of polycrystalline film with large grains in a pristine state of the film and under positive and negative voltage application.

Temperature induced variation of the *I*–*V* characteristics measured at 30–295 K indicates that the power dependence $I{\sim }U^n$ is observed in a whole temperature range: with $n=3$ at 70–295 K, and $n>3$ at lower temperatures, see Fig. S6 in Supplementary materials for details. This fact suggests the prevalence of TAT influenced by SCLC as a dominant transport mechanism at all available temperatures. According to the cycling STS measurements carried out at different temperatures, cooling down the sample, which exhibits a gradual change of the resistance state, reduces the step between neighboring *I*–*V* curves (Fig. 6a,b). We associate such T-dynamics with oxygen vacancies redistribution in polycrystalline film due to their migration in the applied electric field. This is schematically illustrated in Fig. 6c.

In the pristine state of the film, the oxygen vacancies are mainly concentrated at the interface with the bottom electrode of the structure (Fig. 6c), screening the built-in field, which favored upward direction of the polarization in the polycrystalline sample with large grains. If we assume trap-assisted tunneling to be a dominant transport mechanism and expect electron injection to occur from the bottom electrode, an enhanced concentration of oxygen vacancies at the interface with the bottom electrode will provide smaller length of hops between the traps near the injecting contact. Thus, the pristine resistive state of polycrystalline FE film has the highest conductivity, see Fig. 6a, first voltage sweep (red star). The application of a negative voltage to the TE does not change the polarization orientation but redistributes the oxygen vacancies over the film thickness (Fig. 6c, yellow star). This, in turn, increases the length of the hop and decreases the hopping conductivity. Sequential positive voltage application leads to the polarization reversal. A new polarization state is downward and the electron injection from the bottom electrode reduces the number of positively charged traps by charge trapping (Fig. 6c, white star). We would like to emphasize here the time scale difference for electronic (electronic trapping) and ionic (migration of the charge oxygen vacancies) processes. Together with a back-diffusion, this difference results in another oxygen vacancy distribution after the reversed voltage polarity application. Therefore, the second voltage sweep starts with the modified distribution of the oxygen vacancies in FE film and higher value of resistivity associated with the increased hopping length (Fig. 6c, green star). This assumption is supported experimentally by the *I*–*V* curves measured at room temperature (Fig. 6a): the higher the number of switching cycles, the lower the conductivity of polycrystalline BTO film. Taking into account the temperature dependence of oxygen vacancy mobility, its redistribution is expected to be significantly diminished while cooling the sample. Indeed, cycling measurements at 30 K prove the lowering of the step between two neighbouring *I*–*V* curves with decreasing temperature (Fig. 6b). To cover the range between “the most resistive” and “the most conductive” states, one need ten times more voltage sweep cycles at 30 K than at room temperature.

We attribute the difference in the resistive properties of polycrystalline FE films with distinct grain sizes to the varying contribution of the electronic and ionic processes. The former implies a hopping transport with variable-length jumps, while the latter involves ionic vacancies migration under a bias voltage with regard to the built-in field. The dimension of crystallites determines (1) the density of point defects related to the density of grain boundaries, and (2) the distribution of FE properties over the grains. The specificity of polarization charge screening at the interfaces leads to the nonuniform distribution of the charged point defects over the film thickness, thus influencing the impact of the ionic transport on the resistive properties of FE film.

In summary, epitaxial, nanocrystalline and polycrystalline BTO thin films with different sizes of grains were grown to reveal the influence of the film structure on the local electric and ferroelectric properties. For polycrystalline film with large grains in its pristine state, the peculiarity of screening the built-in field by positively charged oxygen vacancies defines the predominant polarization orientation. The prevailing transport mechanism is the trap-assisted inelastic tunneling (traps are represented by the oxygen vacancies). An impact of oxygen vacancies redistribution over the film thickness under sequent voltage application modifies the resistive properties of polycrystalline FE films due to competing processes of electron injection, trap filling and drift of the positively charge oxygen vacancies under the applied electric field. Our study is expected to be an important step towards comprehensive understanding of the interplay of depolarizing and built-in fields with a specificity of charge transport mechanisms in thin FE films.

## Materials and methods

LSMO/BTO heterostructures were deposited on a (001)-oriented single-crystalline STO substrates. Commercially available SrTiO$_{3}$ STEP substrates (Shinkosha) having an excellent polished surface with flat atomic/molecular step terraces have been used in this study. The LSMO (10 nm)/BTO (4–11 nm) heterostructures were grown via PLD on the above mentioned substrates. It should be noted, that one of the strength of PLD lies on its effective conveyance of cation stoichiometry from target to the substrates, while oxygen stoichiometry depends on the growth parameters. From this point of view, we consider that traps in our BTO thin films are mainly point defects that create vacancies in anion sub-lattice and could be associated with oxygen vacancies^26–28^. After deposition, the samples were cooled down to room temperature under higher oxygen pressure. Gold TEs with thicknesses of 10–15 nm were deposited on the BTO films by thermal evaporation.

The samples chosen for the studies are 4 nm-thick epitaxial film, 7 nm-thick nanocrystalline film, 7 nm- and 11 nm-thick polycrystalline films with different grain sizes. Epitaxial film, being a reference for the local electric and FE properties, has to be thick enough for eliminating the elastic tunneling as a prevalence transport mechanism, while thin enough to supply measurements in STM regime. The thickness of remaining FE films was chosen to be approximately same, ensuring comparability of their properties with neglecting effect of thickness. The sample with largest grains is thicker than other polycrystalline films, since there is a deviation in height of individual grains from the mean thickness of the film.

The TEs were patterned using a stencil mask, whose areas are in the 2 $\upmu \mathrm{m}^2$ to 36 $\upmu \mathrm{m}^2$ range. For epitaxial BTO films, X-ray diffraction (XRD) experiments are carried out with a SmartLab difractometer (Rigaku) equipped with a 9 kW Cu anode X-ray tube. The results of the in-plane XRD scan confirmed that the LSMO and the BTO films are fully strained on the STO substrate since the in-plane lattice of the BTO film equals the in-plane lattice of the STO substrate. The growth conditions (for all samples) together with X-ray theta-2theta scans and in-plane mapping (for epitaxial thin films) are summarized in Supplementary materials, see Table S1, Figs. S1 and S2.

The ferroelectric properties of fabricated heterostructures were measured at room temperature in ambient, employing piezoresponse force microscopy (PFM) technique of a Veeco SPM instrument. Commercially available Pt-coated silicon tips were employed for the PFM measurements. Voltage was applied to the bottom electrode of the structure with the probe grounded.

The tunneling AFM and STM/STS measurements were performed under ultra-high vacuum (UHV) conditions at the resource center “Physical Methods of Surface Investigation” (RC PMSI) of Research Park of Saint Petersburg State University. The base pressure in the UHV chamber was better than $3\times 10^{-10}$ mbar. Residual gas particles adsorbed on a sample surface were removed by a heating up to 120$^{\circ }{\mathrm{C}}$ in UHV before measurements. The *I*–*V* curves were obtained in a tunneling current range from 1 pA to 330 nA at the variable sample temperature in the range from 30 K to 295 K using an Omicron VT AFM XA scanning probe microscope. STM and AFM data processing and analysis were performed with Gwyddion software^29^.

## Supplementary information

Supplementary Information.
