# Supplementary material for: Structure-resistive property relationships in thin ferroelectric BaTiO3 films
Source: Sci Rep. 2020 Sep 28;10:15848. doi: 10.1038/s41598-020-72738-5 (PMC7522979; doi:10.1038/s41598-020-72738-5)
Supplement: Supplementary file 1 — Supplementary Information. [file 41598_2020_72738_MOESM1_ESM.pdf]

SUPPLEMENTARY INFORMATION

Structure–resistive property relationships in thin ferroelectric $BaTiO_3$ films

N. V. Andreeva[1,*], A. Petraru[2], O. Yu. Vilkov[3], and A. E. Petukhov[4]

[1]St. Petersburg Electrotechnical University ‘LETI’, Saint Petersburg, 197376, Russia
[2]Nanoelektronik, Technische Fakultät, Christian-Albrechts-Universität zu Kiel, D-24143 Kiel, Germany
[3]Department of Solid State Electronics, St. Petersburg State University, Saint Petersburg, 198504, Russia
[4]Research Park, St. Petersburg State University, Saint Petersburg, 198504, Russia
[*]nvandr@gmail.com

On the growth conditions and preliminary characterization of the samples.
For this study more than 30 $SrTiO_3/La_{0.7}Sr_{0.3}MnO_3/BaTiO_3$ (STO/LSMO/BTO) heterostructures were grown. The growth conditions for all samples are summarized in the Table S1 below. Samples chosen for the paper are highlighted in red.

Sample	Growth conditions of BTO films						Growth conditions of bottom LSMO electrodes				
	T^{FE}, °C	P_{gr}^{FE}, mbar	N^{FE} Pulses	E^{FE}, J/cm^2	f^{FE}, Hz	P_{an}^{FE}, mbar	T^{BE}, °C	P_{gr}^{BE}, mbar	N^{BE} Pulses	E^{BE}, J/cm^2	f^{BE}, Hz
slb161123	800	0.34	100	1.5	1	1	800	0.34	250	1.5–1.7	1
slb161124	600	0.1	100	1.5	1	1	750	0.164	200	1.5	1
slb161125	550	0.1	50	1.5	1	1	750	0.32	250	1.5	1
slb161130	650	0.2	50	2.0	1	1	750	0.16	150	2.0	2
slb161201	550	0.2	40	1.7	1	0.38	750	0.2	250	1.7	2
slb161202	550	0.2	30	1.7	1	0.4	750	0.2	200	1.7	1
slb161205	500		30	1.1	1	0.4	750	0.2	200	1.8	2
slb161206	500		30	1.1	1	0.4	750	0.2	250	1.1	1
slb161208	500		40	1.1	1	0.4	500		80	1.1	1
slb161209	500	0.1	40	1.1	1	0.4	750	0.2	250	1.8	1
slb161212	500	0.1	40	1.1	1	0.4	750	0.2	200	1.1	1
slb170228	500		40	1.1	1	0.4	750	0.2	200	1.3	2
slb170301	500		25	1.1	2	0.4	750	0.17	200	1.3	2
slb170302	500		20	1.1	1	0.4	750	0.21	250	1.3	2
slb170303	500	0.08	25	1.1	1	0.4	750	0.17	170	1.3	2
slb170303b	500	0.08	25	1.1	1	0.4	750	0.17	150	1.3	2
slb170306	500	0.17	30	1.2	1	0.4	750	0.17	200	1.5	1
slb170307	500		30	1.3	1	1.3	750	0.21	200	1.3	2
slb170308	500		30	1.1	1	0.4	750	0.21	100	1.3	2
slb170309	500	0.1	25	1.1	1	0.4	750	0.21	200	1.3	2
slb170310	500	0.08	45	1.1	1	0.4	750	0.17	150	1.3	2
slb170310b	500	0.1	55	1.1	1	0.4	750	0.17	100	1.3	2
slb170313	500	0.08	45	1.1	1	0.4	750	0.17	170	1.3	2
slb171010	500	0.07	45	1.1	1	3	750	0.2	200	1.7	2
slb171012	500	0.1	40	1.3	1	3	750	0.2	200	1.3	1
slb171013	650	0.1	50	1.1	1	2	750	0.14	200	1.1	2
slb171025	750	0.1	45	2.0	1	1.3	750	0.2	150	2.0	1
slb171026	500	0.1	40	1.3	1	3	750	0.25	225	2.0	2
slb171027	680	0.1	40	1.3	1	1.3	710	0.13	200	1.3	2
slb171030	720	0.08	40	1.3	1	1.3	710	0.1	240	1.3	2

Continued on next page

Sample	Growth conditions of BTO films						Growth conditions of bottom LSMO electrodes				
	T^{FE}, °C	P_{gr}^{FE}, mbar	N^{FE} Pulses	E^{FE}, J/cm^2	f^{FE}, Hz	P_{an}^{FE}, mbar	T^{BE}, °C	P_{gr}^{BE}, mbar	N^{BE} Pulses	E^{BE}, J/cm^2	f^{BE}, Hz
slb171101	700	0.08	45	1.1	1	0.5	720	0.1	240	1.1	2
slb171102	600	0.1	45	1.1	2	1	720	0.1	240	1.1	2
slb171103	580	0.14	45	1.1	1	2.5	750	0.1	110	1.1	1
slb171106	700	0.2	45	1.7	1	1.3	750	0.2	200	1.8	2
slb171108	500	0.1	30	1.1	1	5	750	0.19	200	1.7	1

Table S1. Growth conditions of ferroelectric (FE) films and of the bottom LSMO electrodes: substrate temperature — T^{FE}, T^{BE}; oxygen partial pressure during the films growth — P_{gr}^{FE}, P_{gr}^{BE}, and at the annealing — P_{an}^{FE}; number of laser pulses — N^{FE}, N^{BE}; laser fluence — E^{FE}, E^{BE}; laser repetition rate — f^{BE}, f^{FE}.

The samples from Fig. 2 of the main text were grown on STO(100) STEP substrate. After the deposition, epitaxial BTO films were annealed at temperatures between 500°C and 800°C in 0.4 mbar of oxygen partial pressure for 1 hour. Sample cooling took place at 0.33 mbar of oxygen with a cooling rate of 10°C/min. The sample **slb161209** with epitaxial BTO film is shown in Fig. 2(a, e).

The deposition process was monitored *in situ* with Reflection High-Energy Electron Diffraction (RHEED) operated at high voltage of 20 kV and electron beam current of 38 mA [Fig. S1]. RHEED intensity oscillations indicate a layer-by-layer growth of LSMO. Two RHEED patterns shown in the inset of Fig. S1 were taken at the moments marked by arrows. During the growth of BTO films, no RHEED oscillations were observed, but the RHEED pattern remained that of a two-dimensional surface, indicating changes of the growth mode to a step-flow [1].

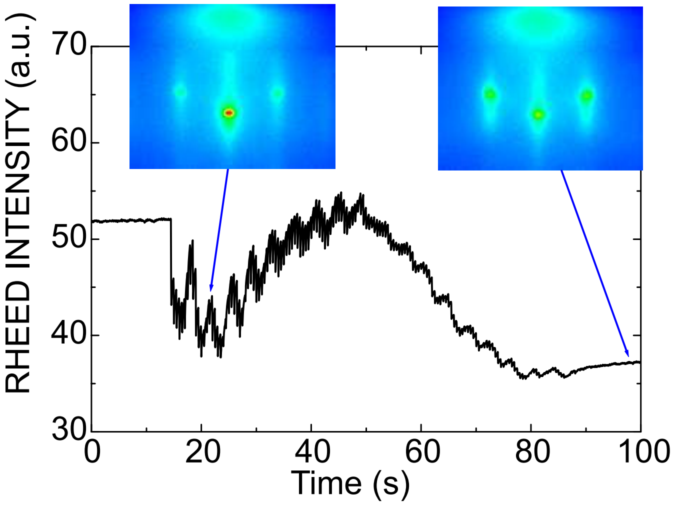

Figure S1. RHEED intensity as a function of time during LSMO deposition. The insets show tho RHEED patterns acquired during LSMO film deposition.

X-ray diffraction (XRD) experiments were carried out with a SmartLab diffractometer (Rigaku) equipped with a 9 kW Cu anode X-ray tube. XRD data of 3.5 nm-thick BTO film grown on LSMO/STO structure are presented in Fig. S2. The normal two theta-omega scan from Fig. S2(a) shows that BTO/LSMO films are epitaxially grown on the (100) STO substrate, BTO films are *c*-axis oriented with the *c* direction perpendicular to the substrate surface, having a *c* lattice parameter of 4.15 Å. The in-plane XRD spectrum was acquired without monochromator due to the weak intensity of the diffraction signal and this explains the presence of the $Cu_{K\beta}$ and $W_{K\alpha 1,2}$ lines observed near the (200) diffraction peak of the BTO film. The spectrum was acquired with a four-bounce monochromator. From the in-plane XRD scan shown in Fig. S2(b) we found the in-plane lattice parameter of $a = 3.91$ Å for BTO films. LSMO and BTO films are fully strained on the STO substrate, since the in-plane lattice parameters of BTO film equals to that of the substrate.

The sample **slb170310** is shown in Fig. 2(b, f). After the deposition, BTO film was annealed at 650°C for 30 min at 0.4 mbar of oxygen partial pressure. Sample cooling took place at 0.4 mbar of oxygen, with a cooling rate of 10°C/min.

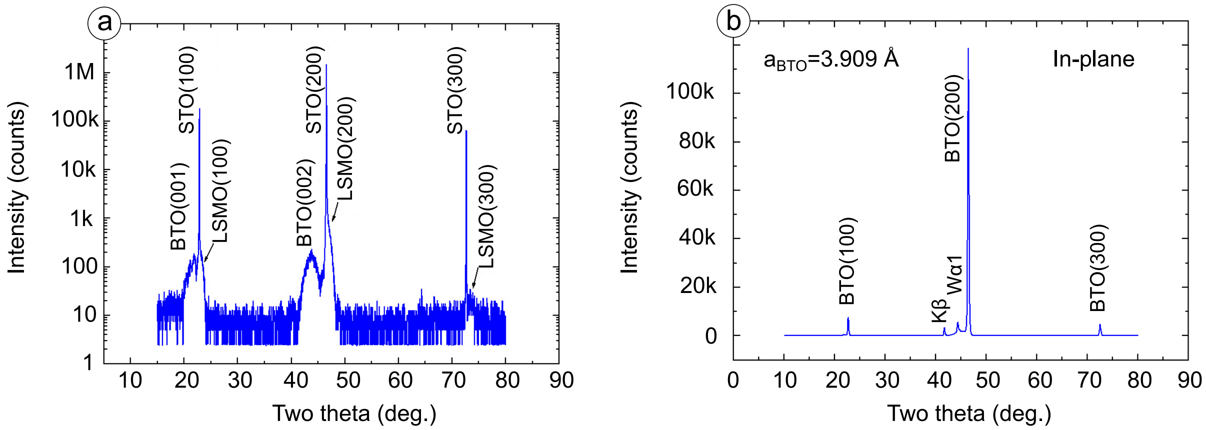

Figure S2. XRD scans of a STO/LSMO/BTO(4 nm) heterostructure: (a) — normal two theta-omega scan measured with monochromatic Cu $K_{\alpha 1}$ radiation; (b) — in-plane scan of the same heterostructure (non-monochromatic x-ray radiation).

The sample **slb171106** is shown in Fig. 2(c, g). After the deposition, BTO film was cooled down to 620°C at 0.4 mbar of oxygen pressure, and down to room temperature at 1.3 mbar of oxygen pressure with a cooling rate of 10°C/min.

The sample **slb161123** is shown in Fig. 2(d, h). After the deposition, BTO film was cooled down at 0.1 mbar of oxygen pressure with a cooling rate of 10°C/min.

On the lateral resolution of STM.

From a macroscopic point of view, according to the s-wave model [2, 3], the lateral resolution of the STM can be estimated as $[2(R+s)[\text{Å}]]^{(1/2)}$, where R is a radius of the tip, s is a distance between the tip and the surface. In the direction of the surface normal (z-axis), the resolution is less than 0.01 nm for Omicron VT SPM XA microscope, as is given in technical information from the manufacturer and has been proven in our laboratory tests. Mentioned approach is applicable, provided that the linear dimensions of the feature under investigation are much bigger than the surface lattice constant, and the tip-sample separation is large enough. Our measurements were carried out at relatively high voltages and close to the possibly lowest currents (tunneling conditions were $I_t = 10$ pA, $V_s = 1.5$ V). The dependence of the current on the distance is exponential: decreasing the set point of the tunnel current by an order of magnitude leads in increasing the tunnel gap by 1 Å. This fact is widely covered in the literature [4, 5] in contrast to the issue of the origin of z(V) dependence. The latter is shown in the Fig. S3 for the case of gold-intercalated graphene being a good example. For comparison, typical tunneling conditions to achieve atomic resolution on graphene are $I_t = 1$ nA, $V_s = 3$–10 mV with the tunnel gap of several angstroms. Summarizing, we can conclude that the approach for estimating the STM lateral resolution is applicable here.

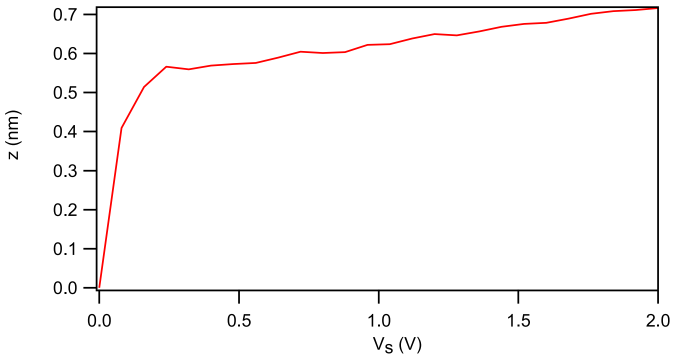

Figure S3. The relative tip-sample displacement versus different bias voltages for graphene/Au/Co(0001).

It should be noted that the terms “high spatial resolution” and “atomic resolution” should not be confused. A special case of high-resolution STM is atomic resolution, which, for most structures, cannot be described by Tersoff-Hamann’s theory. To achieve atomic resolution both the presence of localized electronic states of the tip and localized states of the sample are necessary. Theoretical description of the atomic resolution can be given using the so-called “Chen’s derivative rule” [6]. Radius R was estimated from Scanning Electron Microscope (SEM) image of STM tip obtained in the backscattered electrons detection

mode (AsB) with a beam energy of 20 kV and a beam current of 281 pA. Taking the sum of $R = 75$ Å and $s = 10$ Å to be $R + s = 85$ Å, we can estimate the upper limit of the resolution of about 1 nm. Note, the actual radius of the tip curvature can be much less than the resolution achievable in the SEM.

On the lateral resolution of AFM.
Geometrical analysis of a tip-sample contact [7] allows us to obtain the following expression for the minimal separation between the resolved asperities when the AFM image "dip" can still be detected, *i.e.*, for the vertical resolution limit ΔZ: $d \cong [(8(R+r)\Delta Z)]^{(1/2)}$. Because the best spatial resolution must be the invariant characteristic of the instrument (independent on the studied object), it should be defined, *e.g.*, from the condition of two point objects ($r = 0$) detection. Then, the best lateral resolution limit d for a standard AFM instrument with a vertical resolution limit $\Delta Z = 0.1$ nm and tip (with conductive coating) curvature radius $R = 35$ nm, equals to $d \approx 5$ nm.

On the conductivity of LSMO electrode.
AFM image of LSMO electrode is provided in the Fig. S4. The metallic type of conductivity was validated by measurements of temperature dependence of the film resistivity through the top electrode deposited on the LSMO layer. The resistivity of the LSMO layer at RT is well below 1 $\Omega \cdot cm$. The conductivity map of LSMO electrode surface was also measured with cAFM (diamond coated tips were used) and represented the uniform distribution of current over the surface.

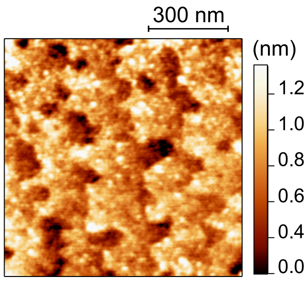

Figure S4. Contact mode AFM images of the LSMO film surface.

On the local ferroelectric properties of the samples.
Piezoresponse phase distribution over the sample surface and FE hysteresis loops were measured for all samples [Fig. S5]. Epitaxial, nanocrystalline and polycrystalline samples with medium grains exhibit a uniform piezoresponse distribution over the surface as well as symmetric and rectangular FE loops. The results of the poling procedure, that is a scan over rectangular regions with DC voltage between the bottom electrode and the grounded conductive tip, reflect the fact, that changing the polarity of the voltage applied gives rise to the corresponding polarization reorientation in the film: bright and dark areas in the phase images in Fig. S5(a—d) are related to the upward or downward directions of polarization, respectively. Typical FE loop for these films are shown in Fig. S5(e, f).

For BTO films with large crystallites, FE domain structure is clearly distinguished inside individual grains in the as-grown state Fig. S5(g, h). Prevalence of the domains with an upward polarization (60 - 70 % of the total number of domains) is observed. A significant contrast in the PFM phase distribution appears only after poling with negative voltage and is attributed to the downward polarization orientation. The image of the regions poled with positive voltage does not differ considerably from that of the pristine state. Together with imprinted local piezoresponse loops, these results justify the preference of an upward polarization orientation in as-grown polycrystalline BTO films with large grains. The mean value of the imprint bias is about 0.4 V for BTO film with large grains. The shape of the local hysteresis loop does not change much at different locations, indicating a uniform distribution of the local FE properties over the film surface [Fig. S5(f)].

On the specificity of local STS measurements of epitaxial and nanocrystalline samples.
STM imaging of epitaxial film requires $V_s = 4.5$ V to ensure $I_t = 1$ pA, while the parameters are 4 V, 10 pA and 1.5 V, 10 pA for nanocrystalline and polycrystalline films, respectively. A simple estimation of the STM probe area involved in the current measurements suggests the value of 1 nm for it. Thus, measurements of the local *I-V* characteristics with STM probe require high current density for epitaxial and nanocrystalline FE films, which provokes a non-reversible modification of the film structure. Indeed, the comparison of the STM images before and after *I-V* curve measurements proves the modification. For polycrystalline FE films, STM experiments are not accompanied by the structure modification.

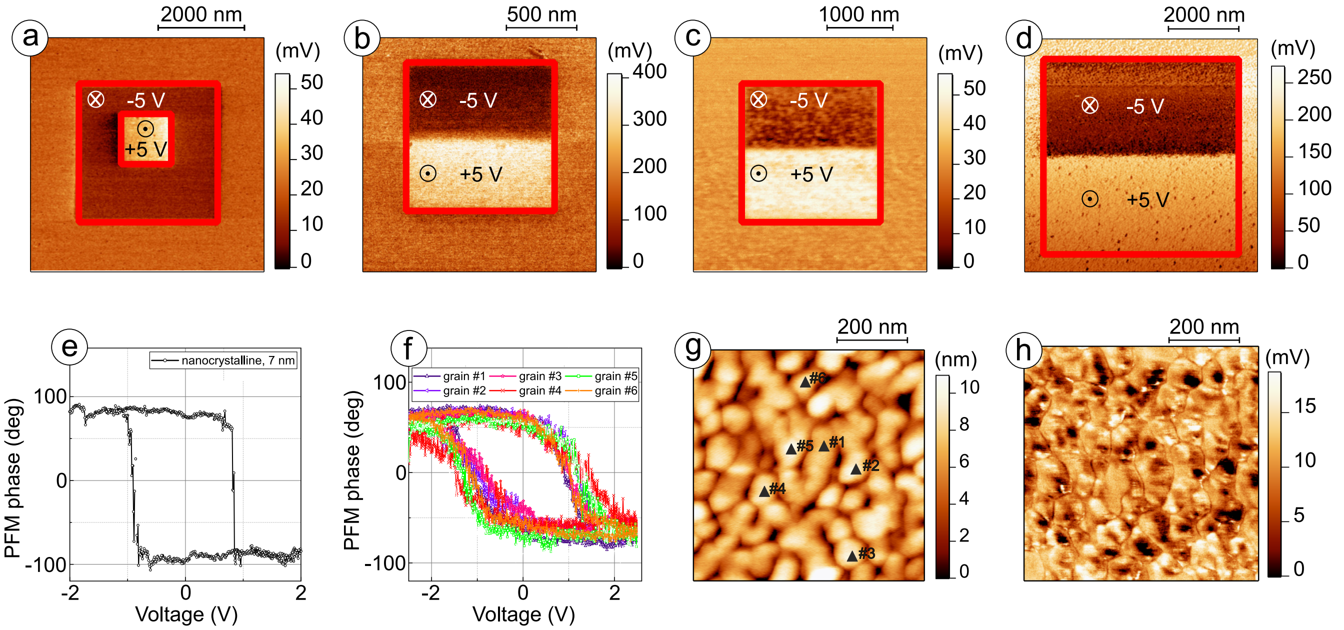

Figure S5. FE properties of STO/LSMO/BTO heterostructures measured with PFM at room temperature in ambient: (a) — PFM phase image after the poling procedure for epitaxial BTO film, square domains with downward and upward polarizations written with ±5 V; (b) — PFM phase image after the poling procedure for nanocrystalline BTO film, square domains with half squares of downward and upward polarizations written with ±5 V; (c) — PFM phase image after the poling procedure for polycrystalline BTO film with medium grain sizes, the square domains with half squares of downward and upward polarizations written with ±5 V; (d) — PFM phase image after the poling procedure for polycrystalline BTO film with large grain sizes, the square domains with half squares of downward and upward polarizations written with ±5 V; (e) — local piezoresponse-voltage hysteresis loops of nanocrystalline BTO films measured on bare film surface in spectroscopic PFM regime; (f) — local piezoresponse-voltage hysteresis loops of polycrystalline BTO film with large grain sizes measured on bare film surface in spectroscopic PFM regime at different locations, specified in (g); (g) — contact mode AFM image of polycrystalline BTO film with large grain sizes; (h) — PFM phase image of domain structure in polycrystalline BTO film with large grain sizes in its pristine state from (g).

On the temperature dynamics of local resistive properties of the polycrystalline samples with medium and large grains. STS *I-V* curve measurements of polycrystalline films with medium and large grains are nonlinear and asymmetric, and agree well with the results of conductive AFM measurements with TEs involved. *I-V* curves demonstrate a pinched hysteresis [Fig. S6(a,b)] that we have previously observed in thin epitaxial BTO films (with thicknesses $d > 4$ nm) in the broad temperature range (30–295 K) [8]. It is partially associated with the impact of the polarization switching currents on the measured current.

For polycrystalline films, asymmetries of the *I-V* curves [Fig. S6(a, b)] and of the FE loops [Fig. S5(f)] do not correlate with each other. Namely, the switching voltage for the the nonlinear part of *I-V* curves is twice smaller than the coercive voltage of FE film (extracted from the local piezoresponse loops) for the forward branch, and exceeds several times the coercive voltage for the reversed branch. This indicates that memristive behavior of thin FE films cannot be fully attributed to the specificity of polarization charge screening as in the case of tunnel electroresistance in FTJs.

It should be noted that *I-V* curves obtained with STM tip are linear in a double logarithmic scale [Fig. S6(c, d)] with characteristics part corresponding to power dependence $I \sim U^n$ with $n = 3$ in the whole temperature range. This is consistent with the results of conductive AFM measurements over the TEs and signifies the contribution of SCLC.

As we consider, the resistive properties of polycrystalline FE films are modified compared to that of epitaxial layers due to the point defects involved in the transport mechanisms. The defects are mainly oxygen vacancies and associated complexes. Taking into account the n-type conductivity in BTO, the electronic band structure of polycrystalline BTO films with in-gap states associated with the oxygen vacancies suggests the formation of a rectifying contact with most commonly used electrode materials (platinum, tungsten, LSMO). This explains the appearance of the asymmetry in I-V curves measured on thin polycrystalline BTO films.

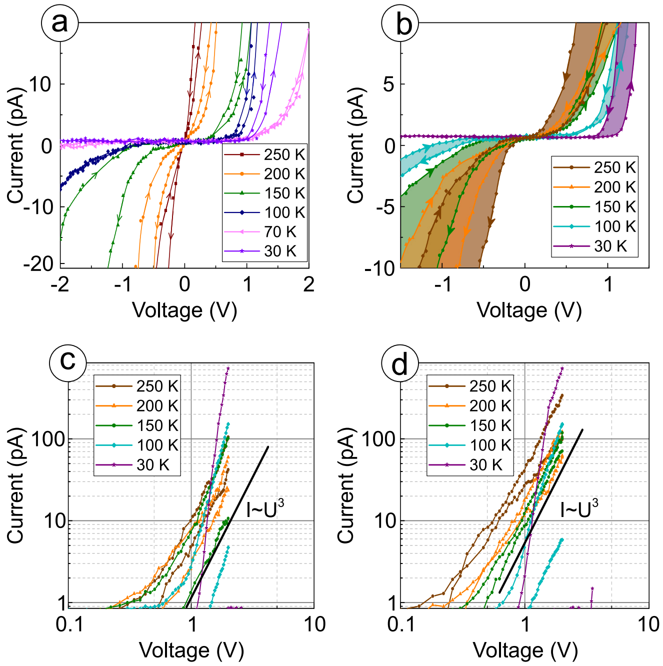

Figure S6. Resistive properties of STO/LSMO/BTO heterostructures in the temperature range of 30–250 K: (a) — local *I-V* characteristics of polycrystalline BTO films with medium grains measured with STS in the temperature range of 30–250 K; (b) — *I-V* characteristics of polycrystalline BTO films with large grains measured with STS in the temperature range of 30–250 K; (c) — *I-V* characteristics for forward branch from (a) plotted in a double logarithmic scale; (d) — *I-V* characteristics for reversed branch from (b) plotted in a double logarithmic scale.

References

1. Choi, J. & Eom, C.B. Growth mode transition from layer by layer to step flow during the growth of heteroepitaxial $SrRuO_3$ on (001) $SrTiO_3$. *Appl. Phys. Lett.* **79**, 1447-1449, DOI: 10.1063/1.1389837 (2001).

2. Tersoff, J. & Hamann, D. Theory and application for the scanning tunneling microscope. *Phys. Rev. Lett.* **50**, 1998–2001, DOI: 10.1103/PhysRevLett.50.1998 (1983).

3. Tersoff, J. & Hamann, D. Theory of the scanning tunneling microscope. *Phys. Rev. B* **31**, 805–813, DOI: 10.1103/PhysRevB.31.805 (1985).

4. Chen, C. *Introduction to Scanning Tunneling Microscopy: Second Edition* (Oxford University Press, Oxford, 2007).

5. Wiesendanger, R. *Scanning Probe Microscopy and Spectroscopy: Methods and Applications* (Cambridge University Press, Cambridge, 1994).

6. Chen, C. Tunneling matrix elements in three-dimensional space: The derivative rule and the sum rule. *Phys. Rev. B* **42**, 8841–8857, DOI: 10.1103/PhysRevB.42.8841 (1990).

7. https://www.ntmdt-si.com/resources/spm-theory/theoretical-background-of-spm.

8. Andreeva, N., Petrov, A., Petraru, A., Petukhov, A. & Rybkin, A. Mechanisms of electron transport in $BaTiO_3$ ultrathin epitaxial films in the temperature range 40 K–295 K. *Mater. Res. Express* **6**, 026427, DOI: 10.1088/2053-1591/aaf24f (2018).
